# Differences and similarities of high-resolution computed tomography features between pneumocystis pneumonia and cytomegalovirus pneumonia in AIDS patients

**DOI:** 10.1186/s40249-020-00768-2

**Published:** 2020-10-26

**Authors:** Chun-Jing Du, Jing-Yuan Liu, Hui Chen, Shuo Yan, Lin Pu, Hao-Feng Xiong, Pan Xiang, Chuan-Sheng Li, Ming Zhang, Ru-Ming Xie, Bu-Dong Chen, Ang Li

**Affiliations:** 1 Department of Critical Care Medicine, Beijing Ditan Hospital, Capital Medical University, No. 8 Jingshundong Street, Chaoyang District, Beijing, 100015 China; 2 Department of Radiology, Beijing Ditan Hospital, Capital Medical University, No. 8 Jingshundong Street, Chaoyang District, Beijing, 100015 China

**Keywords:** Diagnostic imaging, Pneumocystis pneumonia, Cytomegalovirus pneumonia, HIV/AIDS

## Abstract

**Background:**

Accurately differentiating pneumocystis from cytomegalovirus pneumonia is crucial for correct therapy selection in AIDS patients. Hence, the goal of this study was to compare the computerized tomography (CT) features of pneumocystis pneumonia and cytomegalovirus pneumonia in AIDS patients and identify clinical hallmarks to accurately distinguish these two pathologies.

**Methods:**

A total of 112 AIDS patients (78 with pneumocystis pneumonia and 34 cytomegalovirus pneumonia) at Beijing Ditan Hospital from January 2017 to May 2019 were included in this study. Two experienced chest radiologists retrospectively reviewed CT images for 17 features including ground-glass opacity, consolidation, nodules, and halo sign. Binary logistic regression analyses were conducted to identify the significant parameters that distinguished pneumocystis pneumonia from cytomegalovirus pneumonia. Correlations were analyzed by Pearson or Spearman correlation analyses. Result were considered significant if *P* < 0.05.

**Results:**

The presence of consolidation, halo signs, and nodules (all *P* < 0.05) were significantly more frequent in patients with cytomegalovirus pneumonia than in those with pneumocystis pneumonia. Small nodules (32.5% in cytomegalovirus pneumonia, 6.41% in pneumocystis pneumonia, *P* < 0.001) without perilymphatic distribution were particularly common in patients with cytomegalovirus pneumonia. Large nodules were not found in any of patients with cytomegalovirus pneumonia. The presence of ground-glass opacity, reticulation, and bronchial wall thickening (all *P* > 0.05) were common in both groups.

**Conclusions:**

Analysis of consolidation, nodules, and halo signs may contribute to the differential diagnosis of pneumocystis pneumonia or cytomegalovirus pneumonia. However, some CT features considered typical in one or other diseases appear with similar frequency in both cohorts of AIDS patients. CT features are potentially useful for the differential diagnosis of pneumocystis pneumonia and cytomegalovirus pneumonia in AIDS patients.

## Background

AIDS remains one of the most widespread infectious diseases and a major global health concern in resource-poor settings [1, 2]. According to United Nations Programme on HIV/AIDS, an estimated 1.7 million people (1.2–2.2 million) acquired HIV worldwide in 2019, still three times higher than 500 000 milestone set for 2020 [3]. *Pneumocystis jirovecii* pneumonia (PJP) and cytomegalovirus pneumonia (CMV-P) are the most common infectious complications in patients with AIDS [4, 5]. PJP mortality rates approach 10–20% in treated patients and up to 100% in untreated patients [6], while up to 70% of CMV-P patients succumb to disease if diagnosis is delayed [7, 8]. Both types of pneumonia have similar clinical symptoms and signs, including fever, unproductive coughing, hypoxemia, and dyspnea. But therapeutic approaches are drastically different due to intrinsic characteristics of the infectious agents. Thus, identifying the pathogenic agent is crucial for diagnosing and initiating appropriate therapies.

The diagnosis of PJP and CMV-P is usually established with identification of *P. jiroveci* and cytomegalovirus inclusion bodies in lung biopsy or sensitive immunocytochemical methods in bronchoalveolar lavage (BAL) fluid [9–12].

Despite current advances in diagnostic methods, the diagnosis of both types of pneumonia is still challenging. Microbiological confirmation is frequently invasive and difficult to obtain, which delays diagnosis and therapy [13]. Furthermore, the absence of microbiological evidence and negative results of serum antibodies are not reliable evidence to rule out pulmonary infection [14]. Imaging, especially high-resolution computed tomography (HRCT), which is fast, non-invasive, and more specific and sensitive than X-rays in detecting early and small lesions, represents a vital tool for guiding the diagnosis and therapeutic progression in patients with pneumonia [15]. To our knowledge, no study directly compared HRCT features between PJP and CMV-P in AIDS patients. Thus, incorporating the radiographic features into the clinical report may help narrow the differential diagnosis. The purpose of this study was to compare pulmonary HRCT features of PJP and CMV-P in AIDS patients and identify clinical hallmarks to accurately distinguish these two pathologies.

## Methods

### Patients and data collection

The institutional ethics committee of Capital Medical University affiliated Beijing Ditan Hospital approved this study and informed consent was waived because of its retrospective nature. HRCT scans were reviewed in 112 AIDS patients with confirmed PJP and CMV-P treated at Beijing Ditan Hospital from January 2017 to May 2019. All patients received bronchoscopy, performed serological and bacteriological examinations were included, and cases with existing co-infections were excluded from the study. The study comprised 78 patients with PJP and 34 patients with CMV pneumonia. Diagnosis of PJP was confirmed by symptoms of infection (fever, cough with or without sputum, leukocytosis or leukopenia) with identification of *P. jiroveci* by silver stain or Periodic Acid-Schiff stain in a bronchoscopy specimen. CMV-P cases were defined by detailed clinical evidence of infection (fever, cough with or without sputum, leukocytosis or leukopenia) with definitive BAL results of fluorescence quantitative PCR. HRCT images selected were the closest to the date of positive BAL diagnostic.

### CT examinations

The thorax CT examinations were performed on a 256-detector row spiral CT scanner (Philips, iCT, Holland), or a 64-detector row spiral CT scanner (GE, Lightspeed VCT, USA). The patient was in the supine position and CT scans were performed at the end of inspiration. The scanning was set at energy of 120 kV and automatic current based on body weight. Images were obtained at 5 mm thickness and 5 mm interval, with pitch of 1.0 (iCT) or 0.8 (VCT) throughout the chest. The images were reconstructed with high spatial resolution algorithm into 1.0 mm (iCT) or 0.625 mm (VCT) thickness. Images were interviewed at window settings suitable for assessing lung parenchyma (window width: 1000–1500 HU; window level: − 600– − 700 HU) and mediastinum (window width: 400–500 HU; window level: 30–40 HU).

### Interpretation of images

The CT images on the medical diagnosis screen were reviewed independently in a random order by two experienced chest radiologists blinded to patients’ clinical information. Final conclusions on the features were reached by unanimous consensus.

According to glossary of terms for thoracic imaging [16, 17], CT features were categorized as (a) consolidation; (b) ground-glass opacity (GGO); (c) mosaic perfusion; (d) crazy-paving pattern; (e) nodule; (f) tree-in-bud sign (TIB); (g) halo sign; (h) bronchial wall thickening; (i) reticulation; (j) mediastinal /hilar lymph node (LN) enlargement; (k) pleural effusion; (l) cavity; and (m 12 \* roman 11 \* roman) cyst.

GGO appeared as an area of hazy increased opacity without obscuring bronchial and vascular margins. Consolidation appeared as an area of increased attenuation that obscured the margins of underlying vascular. When consolidation and GGO were both presented in an image, the predominance of the consolidation or GGO (Cons/GGO predominance) was recorded as consolidation predominance, GGO predominance, or equal predominance; and their distribution was classified as segmental, non-segmental, or lobular. The crazy-paving pattern appeared as any superimposition of the intralobular interstitial or interlobular thickening within the GGO. The mosaic pattern was defined as patchwork of regions with heterogeneous attenuation. Nodules were defined as a rounded or irregular opacity with well or poorly defined morphology. The size of nodules was classified as micro (< 3 mm), small (3–10 mm), or large (> 10 mm) and their distribution were recorded as perilymphatic, centrilobular, or random. The tree-in-bud sign (TIB) represented centrilobular branching structures that resemble a budding tree. The halo sign was a GGO surrounding a nodule or mass.

### Statistical analysis

Data were analyzed using SPSS 19.0 software package (IBM, Chicago IL, USA). Normal distributed variables were analyzed with Student’s *t*-test. Non-normal distributed variables were compared by Kruskal–Wallis test. Categorical variables were analyzed by the Fisher’s exact test and the chi-square (*χ*^2^) test. Binary logistic regression analyses were conducted to identify the significant parameters that distinguished PJP from CMV-P. Correlations were analyzed by Pearson or Spearman correlation analyses. Result were considered significant if *P* < 0.05.

## Results

### Baseline characteristics of the patients

Patients demographics and clinical settings are shown in Table 1. No differences in age, gender, weight, and transmission route (*P* > 0.05) were found in PJP and CMV-P cohorts. In terms of inflammation, there was no significant difference in leukocyte counts (*P* = 0.22) and C-reactive protein (*P* = 0.75) between both groups, however, the neutrophil percentage in PJP patients was higher than in CMV-P group (*P* = 0.01), and the lymphocyte percentage in CMV-P was higher than in PJP group (*P* = 0.02). In addition, the HIV viral loads were significantly higher in PJP than in CMV-P (*P* = 0.02) while no differences were found in CD4^+^T cell counts (*P* = 0.28), CD8^+^ T cell counts (*P* = 0.54), and CD4/CD8 ratio (*P* = 0.48) between two cohorts.

**Table 1:** Characteristics of AIDS patients with *Pneumocystis jirovecii* pneumonia and cytomegalovirus pneumonia

	PJP (*n* = 78)	CMV-P (*n* = 34)	*P*-value
Age (years), median (IQR)	33 (29, 45)	38 (29, 48)	0.12
Male, *n* (%)	75 (96.15)	33 (97.06)	0.81
Weight (kg), mean(SD)	64.05 (11.20)	62.82 (9.99)	0.58
Transmission route, *n* (%)			0.41
Homosexual	25 (32.05)	12 (35.29)	
Heterosexual	9 (11.54)	1 (2.94)	
Intravenous drug	1 (1.28)	0	
Blood transfusion	3 (3.85)	1 (2.94)	
Unknown	40 (51.28)	20 (58.82)	
Leukocyte count (× 10^9^/L), median (IQR)	5.50 (3.64, 8.57)	4.52 (3.20, 6.85)	0.22
Neutrophil percentage(%), median (IQR)	79.39 (61.08, 86.42)	68.15 (56.18, 79.50)	0.01
Lymphocyte percentage (%), median (IQR)	11.91 (8.48, 19.54)	18.20 (12.10, 27.24)	0.02
CRP (mg/L), median (IQR)	12.65 (3.40, 36.23)	13.30 (2.70, 35.23)	0.75
HIV viral load log_10_ (copy/ml), median (IQR)	5.50 (5.17, 5.93)	5.19 (4.51, 5.67)	0.02
CD4 T cell count (cells/μl), median (IQR)	22 (9, 47.75)	22.50 (13, 77)	0.28
CD8 T cell count (cells/μl), median (IQR)	458 (329.75, 679)	485.50 (323.75, 779.50)	0.54
CD4/CD8 ratio (%), median (IQR)	0.05 (0.02, 0.10)	0.06 (0.02, 0.16)	0.48

*PJP* *Pneumocystis jirovecii* pneumonia, *CMV-P* cytomegalovirus pneumonia, *CRP *C-reactive protein, *IQR* interquartile range, *SD* standard deviation

### CT features between PJP and CMV-P

**Fig. 1:**
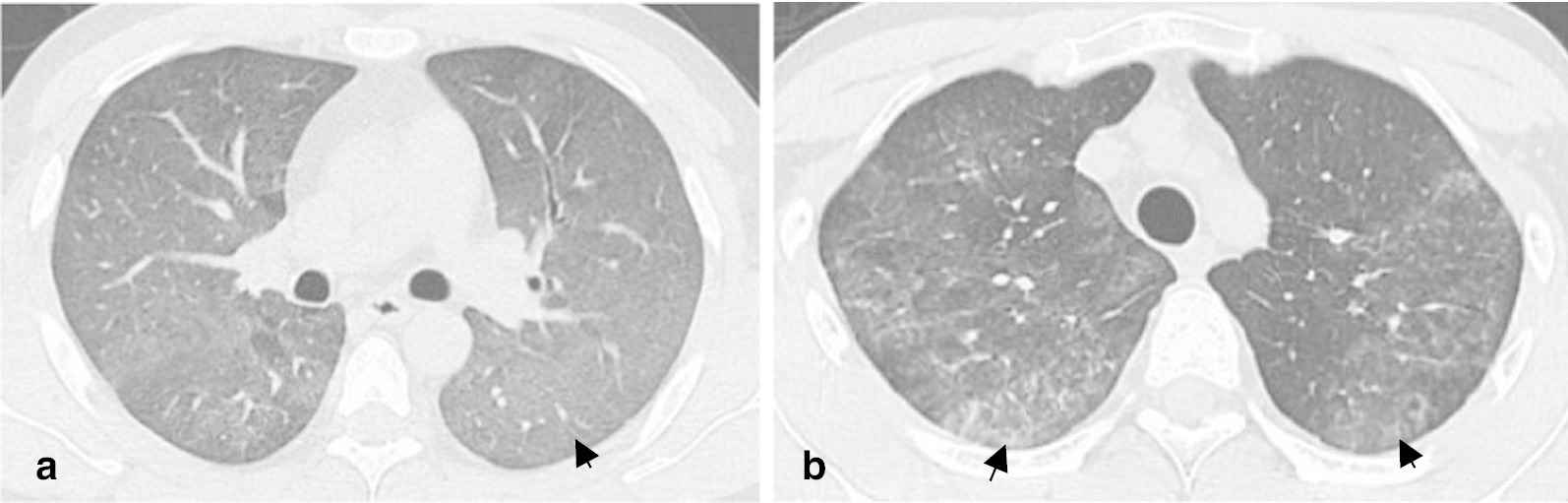
*Pneumocystis jirovecii* pneumonia. **a** Pneumonia caused by *P. jirovecii* in a 28-year-old male patient with AIDS. HRCT image showed diffuse ground-glass opacity of bilateral pulmonary parenchyma (arrow). **b** Pneumonia caused by *P. jirovecii* in a 31-year-old male patient with AIDS. HRCT image showed multiple ground-glass opacity of bilateral pulmonary parenchyma with reticulate changes (arrows)

**Fig. 2:**
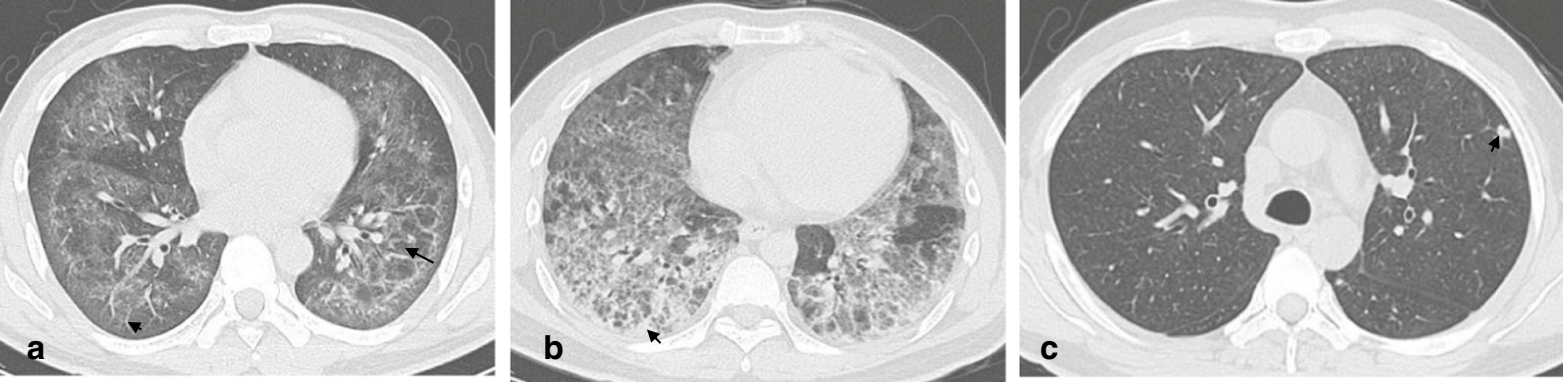
Cytomegalovirus pneumonia. **a** Pneumonia caused by cytomegalovirus in a 38-year-old male patient with AIDS. HRCT image showed ground-glass opacity, reticulation (arrow), and mosaic perfusion (long arrow). **b** Pneumonia caused by cytomegalovirus in a 36-year-old male patient with AIDS. HRCT image showed ground-glass opacity, reticulation, consolidation (arrow), and mosaic perfusion. **c** Pneumonia caused by cytomegalovirus in a 29-year-old male patient with AIDS. HRCT image showed ground-glass opacity, nodules with halo sign (arrow) and tree in bud sign

Thoracic CT features of the 112 patients are shown in Tables 2 and 3. There was no significant difference between PJP and CMV-P in the time between clinical onset and CT examination (6 days in PJP and 5 days in CMV-P, *P* = 0.99), as well as the time between CT and bronchoscopy (5 days in PJP and 3.50 days in CMV-P, *P* = 0.07). GGO was common in AIDS patients with PJP and CMV-P (Figs. 1a and 2a), with 100% penetrance in both cohorts. Contrastingly, the frequency of cavity was rare in PJP (2.56%) and CMV-P (0%). No significant differences were found in mosaic perfusion, crazy-paving pattern, TIB, bronchial wall thickening, reticulation, LN enlargement, cavity, and cyst between both cohorts (all *P* > 0.05). As shown in Table 3, the frequency of consolidation and nodules were significantly higher in patients with CMV-P than in those with PJP (*P* = 0.01 and *P* < 0.001, respectively) (Fig. 1a, b and Fig. 2b, c). Compared to patients with PJP, large nodules with perilymphatic distribution were not found in any of patients with CMV-P. In addition, halo sign was more frequent in CMV-P (32.35%) than in PJP (11.54%). To identify the significant parameters that distinguished PJP from CMV-P, binary logistic regression analyses were conducted and 2 significant parameters were identified: consolidation (*P* = 0.020; *OR*: 3.015; 95% *CI*: 1.190–7.637) and nodules (*P* < 0.001; *OR*: 9.298; 95% *CI*: 3.191–27.095) (Table 4). Combining consolidation and nodules, the AUC for distinguishing PJP from CMV-P was 0.769.

**Table 2:** Thoracic CT features without significant differently frequency

	PJP (*n* = 78)	CMV-P (*n* = 34)	*P*-value
Time between clinical onset and CT examination (days), median (IQR)	6 (3, 10)	5 (3, 13.25)	0.99
Time between CT and bronchoscopy (days), median (IQR)	5 (3, 7)	3.50 (2, 6)	0.07
GGO, *n* (%)	100	100	NS
Mosaic perfusion, *n* (%)	25 (32.05)	10 (29.41)	0.78
Crazy-paving pattern, *n* (%)	7 (8.97)	3 (8.82)	0.98
Cons/GGO predominance, *n* (%)			0.82
Cons	2 (2.56)	2(5.88)	
GGO	75 (96.15)	32 (94.12)	
Equal	1 (1.28)	0	
Cons/GGO distribution, *n* (%)			0.06
Segmental	6 (7.69)	5 (14.71)	
Non-segmental	6 (7.69)	7 (20.59)	
Lobular	66 (84.62)	22 (64.71)	
TIB, *n* (%)	5 (6.41)	5 (14.71)	0.16
Bronchial wall thickening, *n* (%)	27 (34.62)	16 (47.06)	0.21
Reticulation, *n* (%)	45 (57.69)	18 (52.94)	0.64
LN enlargement, *n* (%)	5 (6.41)	5 (14.71)	0.16
Pleural effusion, *n* (%)	8 (10.26)	8 (23.53)	0.07
Cavity, *n* (%)	2 (2.56)	0	0.35
Cyst, *n* (%)	13 (16.67)	4 (11.76)	0.51

*IQR* interquartile range, *PJP* *Pneumocystis jirovecii* pneumonia, *CMV-P* cytomegalovirus pneumonia, *GGO* ground-glass opacity, *Cons* consolidation, *TIB* tree-in-bud sign, *LN* lymph node, *NS* no significance

**Table 3:** Thoracic CT features with significant differentiating frequency

	PJP (*n* = 78)	CMV-P (*n* = 34)	*P*-value
Nodule, *n* (%)	7 (8.97)	16 (47.06)	< 0.001
Nodule-size, *n* (%)			< 0.001
Micro	1 (1.28)	5 (14.71)	
Small	5 (6.41)	11 (32.35)	
Large	1 (1.28)	0	
Nodule-distribution, *n* (%)			< 0.001
Centrilobular	3 (3.85)	8 (23.53)	
Perilymphatic	1 (1.28)	0	
Random	3 (3.85)	8(23.53)	
Consolidation, *n* (%)	28 (35.90)	21 (61.77)	0.01
Halo sign, *n* (%)	9 (11.54)	11 (32.35)	0.01

Data are presented as numbers of patients, with percentages in parentheses

*PJP* *Pneumocystis jirovecii* pneumonia, *CMV-P* cytomegalovirus pneumonia

**Table 4:** The results of binary logistic regression analysis

HRCT features	B value	Wald value	Odds ratio	*P*-value	95% *Cl*	AUC
Nodules	2.230	16.696	9.298	0.001	3.191–27.095	
Consolidation	1.104	5.417	3.015	0.020	1.190–7.637	0.769
constant	− 1.919	24.951	0.147	0.001		

*HRCT* high-resolution computed tomography, *CI* confidence intervals, *AUC* area under the receiver operating

### Correlation analysis of nodules’ occurrence between PJP and CMV-P patients

We next performed correlation analysis of nodules’ and consolidations’ occurrence between PJP and CMV-P patients (Table 5). Results showed that nodules’ occurrence were positively correlated with CD4^+^T cell count, CD8^+^T cell count and CD4/CD8 ratio, while no significant correlations were found with CRP, neutrophil percentage and HIV viral load in CMV-P patients. Among patients with PJP, no significant correlations were observed between nodules’ occurrence or any other clinical indicators. Similarly, there were no significant correlations between consolidations’ occurrence and other clinical indicators in PJP and CMV-P patients (Additional file 1: Table S1).

**Table 5:** Correlation analysis of nodules’ occurrence between pneumocystis pneumonia and cytomegalovirus pneumonia patients

Variable	PJP (*n* = 78)		CMV-P (*n* = 34)	
	R-value	*P*-value	R-value	*P*-value
CD4^+^T cell count	0.159	0.163	0.545	0.001
CD8^+^T cell count	0.111	0.332	0.392	0.022
CD4/CD8 ratio	0.104	0.366	0.503	0.002
CRP	− 0.059	0.607	0.182	0.304
Neutrophil percentage	0.02	0.859	− 0.233	0.186
HIV viral load log_10_	0.08	0.488	− 0.27	0.122

*PJP* *Pneumocystis jirovecii* pneumonia, *CMV-P* pneumocystis pneumonia, *CRP* C-reactive protein

## Discussion

PJP and CMV-P are two of the most common infectious pathogens in AIDS patients leading to life-threatening pneumonia. In terms of baseline characteristics of enrolled patients, this study shows that CD4 T lymphocyte counts of PJP group and CMV-P group were both lower than 50 cells/mm^3^, highlighting the importance of cell-mediated immunodeficiency for the development of PJP and CMV-P. Here neutrophil percentage in PJP was higher than CMV-P. These may be correlated with an increase of inflammatory cytokines and use of corticosteroid in patients with PJP, that might promote neutrophils recruitment and granulopoiesis [18]. Additionally, lymphocyte percentage in CMV-P patients was higher than that of PJP, which may result from higher HIV viral loads in CMV-P group than that in PJP group.

Here we observed several similarities in radiological presentations between two types of pneumonia. The frequency of GGO were highly common, while the occurrence of cavity was relatively rare in both cohorts. Furthermore, both PJP and CMV-P patients showed similar frequency of reticulation, bronchial wall thickening, mosaic perfusion, cyst, crazy-paving pattern, TIB, and LN enlargement. Diffuse alveolar damage might be the main reason for these similarities [19, 20].

In contrast, the occurrence of consolidation, nodules, and the halo sign was more common in CMV-P than in PJP patients. In addition, large nodules with perilymphatic distribution were not found in any of CMV-P patients. The nodules’ occurrence were positively correlated with CD4^+^T cell count, CD8^+^T cell count and CD4/CD8 ratio in CMV-P patient, while no significant correlations were found in PJP patients. These differences in radiological presentations might result from intrinsic virulence factors and immune responses to these pathogens. Human cytomegalovirus belongs to the herpesvirus subfamily, with seroprevalence of 40–100% in the world’s population [5, 21]. Cytomegalovirus can establish life-long latent infection and reactivate during episodes of immunosuppression [22]. Despite eliciting strong and long-lasting immune responses, these antiviral immune responses cannot clear the virus entirely, nor can they effectively prevent superinfection with additional cytomegalovirus strains or reactivation of the persisting viruses. Hence, alterations in host immunity, such as HIV co-infection, tend to allow for increased virus replication and disease progression [5]. *P. jirovecii* is an extracellular fungus that almost exclusively inhabits alveolar spaces [23], causing pulmonary infection through adhesion to the surface of alveolar epithelium cells (type I pneumocytes) [24]. A normal immune response is sufficient to clear *P. jirovecii* infection in most people without any evidence of latent infection [23]. Moreover, *P. jirovecii* has a unique tropism for the lung, where it exists primarily as an alveolar pathogen without systemic dissemination. Therefore *P. jirovecii* adherence to alveolar epithelium cannot justify such diffuse alveolar damage observed during severe pneumonia. Rather, the patient’s inflammatory response is the primary driver of such extensive alveolar-capillary surface damage [25]. Granulomas are rare in patients with *P. jirovecii* infection, but have been described before [26]. Taken together, these mechanisms induce alterations in host histopathology, which in turn allow for radiological differences between these two diseases.

There are several limitations in present study. The single-centre design may hamper the generalization of our conclusions. Patients included were in different stages of diseases, since BAL results was used as diagnostic criteria, which might influence the frequency of CT features, such as halo signs and cavities; and the lack of histological specimens limited the analysis of correlation with pathologic radiology. In addition, sensitivity and specificity of these patterns in AIDS patients requires further validation in a blinded retrospective or future prospective study.

## Conclusions

The occurrence of consolidation, micro and small nodules with centrilobular or random distribution, and halo sign were highly suggestive of CMV-P rather than PJP in AIDS patients. The GGO, reticulation, and bronchial wall thickening were found with similar occurrence in both pneumonia and, hence, cannot distinguish these two diseases. CT features are potentially useful for the differential diagnosis of pneumocystis pneumonia and cytomegalovirus pneumonia in AIDS patients.

## Supplementary information

**Additional file 1: Table S1.** Correlation analysis of consolidations’ occurrence between pneumocystis pneumonia and cytomegalovirus pneumonia patients.

## Data Availability

Data of this study can be available upon request from the author.

## Abbreviations

AIDS: Acquired immunodeficiency syndrome
AUC: Area under the receiver operating
BAL: Bronchoalveolar lavage
*CI*: Confidence intervals
CMV-P: Cytomegalovirus pneumonia
GGO: Ground-glass opacity
HRCT: High-resolution computed tomography
LN: Lymph node
PCP: Pneumocystis pneumonia
PJP: *Pneumocystis jirovecii* Pneumonia
TIB: Tree-in-bud sign
