# Supplementary material for: Differences and similarities of high-resolution computed tomography features between pneumocystis pneumonia and cytomegalovirus pneumonia in AIDS patients
Source: Infect Dis Poverty. 2020 Oct 26;9:149. doi: 10.1186/s40249-020-00768-2 (PMC7586649; doi:10.1186/s40249-020-00768-2)
Supplement: Supplementary file 1 — Additional file 1: Table S1. Correlation analysis of consolidations’ occurrence between pneumocystis pneumonia and cytomegalovirus pneumonia patients. [file 40249_2020_768_MOESM1_ESM.docx]

Additional file 1: Table S1. Correlation analysis of consolidations’ occurrence between pneumocystis pneumonia and cytomegalovirus pneumonia patients.

|  | PJP (*n* = 78) | | CMV-P (*n* = 34) | |
| --- | --- | --- | --- | --- |
| Variable | R-value | *P*-value | R-value | *P*-value |
| CD4^+^T cell count | -0.106 | 0.355 | -0.204 | 0.247 |
| CD8^+^T cell count | 0.025 | 0.826 | -0.009 | 0.958 |
| CD4/CD8 ratio | -0.135 | 0.239 | -0.210 | 0.234 |
| CRP | 0.172 | 0.131 | 0.191 | 0.278 |
| Neutrophil percentage | 0.063 | 0.581 | -0.053 | 0.764 |
| HIV viral load log_10_ | -0.071 | 0.539 | -0.189 | 0.284 |

PJP = *Pneumocystis jirovecii* pneumonia, CMV-P = pneumocystis pneumonia, CRP = C-reactive protein
